# Retraction Note: Propylthiouracil prevents cutaneous and pulmonary fibrosis in the reactive oxygen species murine model of systemic sclerosis

**DOI:** 10.1186/s13075-022-02973-w

**Published:** 2022-12-14

**Authors:** Gianluca Bagnato, Alessandra Bitto, Natasha Irrera, Gabriele Pizzino, Donatella Sangari, Maurizio Cinquegrani, William Neal Roberts, Marco Atteritano, Domenica Altavilla, Francesco Squadrito, Gianfilippo Bagnato, Antonino Saitta

**Affiliations:** 1 Department of Clinical and Experimental Medicine, Division of Internal Medicine, University of Messina, Via Consolare Valeria n°1, 98100 Messina, Italy; 2 Department of Clinical and Experimental Medicine, Division of Pharmacology, University of Messina, Via Consolare Valeria n°1, 98100 Messina, Italy; 3 Department of Clinical and Experimental Medicine, Division of Rheumatology, University of Messina, Via Consolare Valeria n°1, 98100 Messina, Italy; 4 Department of Internal Medicine, Division of Rheumatology, University of Louisville, Louisville, Kentucky KY 40292 USA

**Retraction Note: Arthritis Res Ther 15, R120 (2013)**

**https://doi.org/10.1186/ar4300**

The Editors in Chief have retracted this article. After publication, Fig 2 was corrected because images in it had been mislabelled [1]. However, new concerns emerged regarding the following:

- Partial image overlap in Fig 3A and C;
- Concerns about band similarity within Western blots in Figs 5A,B and 6A,B;
- The actin bands in 5B appear very similar to the ones published earlier in Fig. 5B in [2] by the same author group.

The authors were unable to produce raw data on request. The Editors have lost confidence in the integrity of the data in this article. None of the authors agree to this retraction.
